# Evaluating Optical Clock Performance for GNSS Positioning

**DOI:** 10.3390/s23135998

**Published:** 2023-06-28

**Authors:** Enkhtuvshin Boldbaatar, Donald Grant, Suelynn Choy, Safoora Zaminpardaz, Lucas Holden

**Affiliations:** School of Science (Geospatial), RMIT University, Melbourne, VIC 3001, Australia; donald.grant@rmit.edu.au (D.G.); suelynn.choy@rmit.edu.au (S.C.);

**Keywords:** optical clocks, satellite atomic clocks, clock stability analysis, Allan deviation, GNSS, Positioning, Navigation, and Timing (PNT)

## Abstract

Atomic clocks are highly precise timing devices used in numerous Positioning, Navigation, and Timing (PNT) applications on the ground and in outer space. In recent years, however, more precise timing solutions based on optical technology have been introduced as current technology capabilities advance. State-of-the-art optical clocks—predicted to be the next level of their predecessor atomic clocks—have achieved ultimate uncertainty of 1 × 10^−18^ and beyond, which exceeds the best atomic clock’s performance by two orders of magnitude. Hence, the successful development of optical clocks has drawn significant attention in academia and industry to exploit many more opportunities. This paper first provides an overview of the emerging optical clock technology, its current development, and characteristics, followed by a clock stability analysis of some of the successfully developed optical clocks against current Global Navigation Satellite System (GNSS) satellite clocks to discuss the optical clock potentiality in GNSS positioning. The overlapping Allan Deviation (ADEV) method is applied to estimate the satellite clock stability from International GNSS Service (IGS) clock products, whereas the optical clock details are sourced from the existing literature. The findings are (a) the optical clocks are more stable than that of atomic clocks onboard GNSS satellites, though they may require further technological maturity to meet spacecraft payload requirements, and (b) in GNSS positioning, optical clocks could potentially offer less than a 1 mm range error (clock-related) in 30 s and at least 10 times better timing performance after 900 s in contrast to the Galileo satellite atomic clocks—which is determined in this study as the most stable GNSS atomic clock type used in satellite positioning.

## 1. Introduction

Precise timing is fundamental to many technological and scientific applications. In particular, Atomic Frequency Standards (AFS), or atomic clocks, have led to remarkable innovations since their invention in the 1950s. For example, atomic clocks have been playing a significant role in determining the International Atomic Time (TAI), whereby the Coordinate Universal Time (UTC) is referenced with the insertion of a leap second [1,2]. As detailed in [2], there is an ensemble of approximately 450 atomic clocks in 80 different laboratories globally to keep the TAI within an average uncertainty of 2 × 10^−16^ s. Furthermore, various space-grade atomic clocks have been developed and deployed into space to enable GNSS satellite positioning techniques for applications like surveying, aviation, transportation, and agriculture. Chip-scale atomic clocks also have been created and used increasingly in terrestrial navigation for self-driving cars, robots, etc.

Notwithstanding the continuous and successful development of AFS over the last few decades, Optical Frequency Standards (OFS), or optical clocks, have been developed recently and outperformed the best AFS by two orders of magnitude [3,4]. Thenceforth, research and development activities focusing on creating more stable, robust, miniature, and low-cost optical clocks are taking place increasingly to improve the current timing capabilities. To evaluate the timing performance of any clock, including optical clocks, clock frequency stability is considered, which describes how consistent the clock frequencies are over different averaging times. The more stable the clock frequencies, the better the timing performance. ADEV is a statistical measure of frequency stability widely used to assess or compare high-precision clocks. Further details regarding ADEV are provided in the following section.

In PNT, likewise, optical clocks are drawing great attention as GNSS positioning is reliant on precise timing. Typically, Positioning and Navigation are to accurately determine the geographic location of any point and to calculate a suitable route between two locations, respectively. Timing, in contrast, is to provide global standard time scales by synchronising time and frequency standards between different clocks situated around the world [5,6]. GNSS is a widely used PNT technology that consists of four main constellations such as Global Positioning System (GPS), Global’naya Navigatsionnaya Sputnikovaya Sistema (GLONASS), Galileo, and BeiDou [7]. A typical GNSS system consists of three fundamental segments, namely (a) space segment, (b) control segment, and (c) user segment. The GNSS space segment, usually a constellation of satellites, is designed to provide PNT services with the use of artificial satellites orbiting Earth. Each satellite carries several (normally three or four) atomic clocks onboard as the primary source of time and frequency standards [7,8,9].

In Positioning and Navigation, which is the focus of this study, precise timing is crucial because a signal travel time between satellite and receiver multiplied by the speed of light, 299,792,458 m/s, determines the range between them. And using a minimum of four ranges, i.e., receiving signals from a minimum of four different satellites simultaneously, can determine the receiver position and the receiver clock time. But, to achieve an error in a range below 1 m, for example, the timing error must be better than three nanoseconds. Numerous studies have discussed the possibility of optical clocks being employed onboard GNSS satellites [10,11,12,13,14,15,16,17,18]; however, an optical clock is yet to be deployed on a GNSS satellite mission.

Among the studies, refs. [10,11] demonstrated a compact rubidium optical clock (CROC) based on two-photon transition achieving 4 × 10^−13^/$\sqrt{\tau \left(s\right)}$ frequency stability over averaging times (τ) up to 10,000 s, which was attractive meeting the technical requirement of GPS Block III satellites, though its long-term stability after that period was not appealing. Whereas German Aerospace Center (DLR) developed a different set-up of an optical clock named Iodine Modulation Transfer Spectroscopy (MTS) with exceptional short-term stability of approximately 10^−15^ from 1 to 10,000 s, and they advocated the clock for the next generation of Galileo satellites (Kepler) with an inter-satellite link and orbit verification applications [12,17,18]. Further, a miniaturised Strontium (Sr) Lattice optical clock based on bosonic atoms was introduced by [13] with 4.1 × 10^−16^/$\sqrt{\tau \left(s\right)}$, which experienced almost 10^−18^ stability after a couple of hours. Even though the miniaturisation of the clock was insufficient for GNSS satellite criteria, the authors highlighted the clock compactness as a first step towards an optical lattice clock setup in space targeting the International Space Station. Meanwhile, refs. [14,15,16] provided a comprehensive review of the current development of space-grade atomic clocks, including the potential use of optical clocks in the future. But the emphases are mainly on the clock development and operating principles.

In Timing, the current time and frequency standards are mostly synchronised via GNSS satellites between terrestrial reference atomic clocks to disseminate UTC time scales to the world [19]. With optical clocks, they do not yet play a formal role in this process. In metrology, however, optical clocks have been recommended as a new way of defining the unit of time [20,21,22,23], second, which was redefined last time in 1967 using the ground state of the caesium^133^ atomic clock [24,25]. Additionally, the International Bureau of Weights and Measures (BIPM)—an intergovernmental organisation of more than 63 countries as of 2022 providing the SI standard for a system of measurements throughout the world—has provided the secondary representations of the second based on various OFS with different optical transitions [26]. For clarity, it is important to note that timing is not the focus of this study, and therefore limited information is presented in that respect.

In this paper, we overview the current development of emerging optical clocks and discuss the potential improvements optical clocks may bring to GNSS positioning based on the clock stability analysis conducted for the aforementioned optical clocks—Sr Lattice, CROC, and Iodine MTS—in comparison with the current atomic clocks onboard GNSS satellites of the four prominent constellations. The overlapping ADEV method is applied to estimate the satellite clock stability from 24 h of IGS satellite clock products with 30 s intervals prepared by GeoForschungsZentrum Postdam (GFZ) analysis centre for 2022 while the optical clock ADEV values are sourced from the existing literature referenced. The significances of this study, in the authors’ view, are first, the stability of satellite atomic clocks (as of 2022) is comprehensively estimated, which was not conducted or found in the existing literature ever since the recent formation of the fully operational four constellations. Secondly, the potentiality of state-of-the-art optical clocks is discussed explicitly from a GNSS positioning user’s perspective and in the context of the clock errors, which has impacts on positioning observations and estimates.

The paper is structured as follows. An introduction and a synopsis of the development of optical clocks are given first. Next, the paper describes clock characterization including frequency stability measures and relevant noise behaviours. Then, data preparation and methodology are detailed for the clock stability analysis, and thereafter, the relevant results and discussion on optical clock potentiality in GNSS positioning applications are presented. In the end, some conclusions are given.

## 2. Optical Clock Development

**Figure 1:**
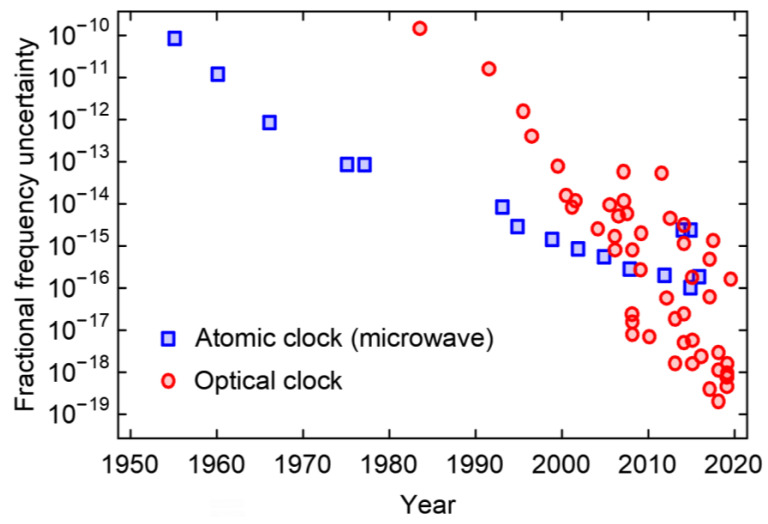
The evolution of AFS and OFS with their fractional frequency uncertainties. The data points shown are referenced from [21].

Since its inception a few decades ago, the revolutionary OFS has significantly improved current timing capabilities and opened up more opportunities. So far, the best optical clock has achieved an uncertainty beyond 10^−18^ and has been recognised as the most stable and accurate timing solution to date [4,27,28,29]. Figure 1 shows the continuous development of AFS and OFS with regard to their fractional frequency uncertainties (ultimate), as summarised in [21].

The principle of optical clock working is very similar to atomic clocks except for the source of oscillation. Replacing the primal oscillator—quartz crystal—with an ultra-stable laser was the key enhancement that amplifies the standard atomic frequencies five orders of magnitude higher, up to 1.1 × 10^15^ Hz [4,30,31]. On the other hand, however, counting such high frequencies (10^15^ Hz) with the current electronic detector was a challenge [32]. In the 2000s, Theodor W. Hänsch and John L. Hall invented an optical frequency comb [33,34], allowing OFS to be measured or converted to microwave frequencies and vice versa. Their remarkable invention of this device was recognised in the industry, and they received the Nobel Prize in Physics in 2005. Optical frequency combs are now the essential component of optical clocks and have contributed significantly to the development of the optical domain. In [34], the authors provide a comprehensive review of optical frequency comb development and applications. In addition, significant improvements in atomic controls and laser stabilisation capabilities have empowered OFS to become more mature [20].

Throughout the development of OFS, neutral atom and trapped ion frequency standards have been improved the most [20] and adopted various elements in the system. For example, optical lattice clocks that require hundreds to thousands of ^87,88^Strontium, ^171,174^Ytterbium, or ^199^Mercury atoms in the system while ^27,40^Aluminum, ^88^Calcium^+^, ^171^Ytterbium^+^, and ^199^Mercury^+^ ions are used in making trapped ion optical clocks [3]. There are other clock types simultaneously developed as well, namely Rubidium two-photon transition and modulation transfer spectroscopy clocks [12].

Making a portable, robust, and miniaturised (chip-scale) optical clock is seemingly the main target in the clock community, besides improving the clock stability performance. For instance, an indication of its transportability was introduced by the authors of [35], who created a ^40^Ca^+^ ion optical clock and achieved 7.3 × 10^−16^ stability over 1000 s, whilst [36] established an ^87^Sr optical lattice clock in a car trailer set-up and showed 4.1 × 10^−17^ stability at a similar averaging time. Lately, Chip-Scale Optical Clock (CSOC) development with integrated photonics has also occurred [37,38,39]. Furthermore, some experiments to test optical clock performance in space scenarios were proposed using a lattice optical clock aboard the International Space Station [40] and a molecular iodine optical clock to be on board the TEXUS 54 sounding rocket to assess the maturity of the clock [41]. This sounding rocket mission was accomplished later in 2018 [42]. Moreover, optical clocks have been recommended for scientific experiments including a test of fundamental physics [4,43], gravity field determination due to their high sensitivity [28,44,45,46,47], a new type of frequency transfer method using a free-space laser link, and fibre optic [4], or via Very Long Baseline Interferometry (VLBI) technique [48]. In addition, Refs. [27,29,49] introduced their successfully developed optical lattice clocks in striving for better timing performance. An extensive review of the evolution of optical clocks can be found in [3,16,20].

## 3. Clock Characterisation

This section presents a brief description of clock characterisation including frequency stability measures and relevant noise behaviours, as these are essential in analysing clock frequency stability. As described in [3], all types of atomic clocks have a similar working principle that requires a source of oscillation to produce stable frequencies. Then, counting the cycles in relation to an initial time will determine the corresponding time. A time output is normally expressed as one pulse-per-second (1 PPS). Although atoms are isolated in a vacuum chamber, their natural frequencies can be perturbed by external environmental effects, normally from electric and magnetic fields and gravity, and therefore the causing errors in the measurement must be characterised in order to correct them. Frequency stability, sometimes referred to as frequency instability, is a critical characteristic of any atomic clock including optical clocks, which describes how consistent the clock frequencies are over a given time interval.

Allan Variance (AVAR) is a standard measure of frequency stability of oscillators used to characterise the clock frequency stability rather than a standard variance due to the non-stationary noise behaviours [16]. AVAR is denoted as $\sigma_{y}^{2}\left(\tau \right)$, where τ is averaging time, also known as integration time or observation time. To measure the frequency stability of a clock, either a single or series of reference clocks is needed. In some cases, clocks were self-tested [49]. In the testing process, the output frequency of the clock under test is compared with reference clock frequency over various averaging times—measured by a reference clock, and the fractional frequency average y over that interval τ is determined. Thus, AVAR is a function of averaging time, τ. The square root of AVAR is ADEV, also known as sigma-tau denoted as $\sigma_{y}\left(\tau \right)$. ADEV is a unitless expression, but it can be read as follows. For example, an ADEV of $\sigma_{y}\left(\tau \right)$ = 1 × 10^−10^ at averaging time τ = 1 s means that there was a fractional frequency change over 1 s. In other words, if $\sigma_{y}\left(\tau \right)$ = 1 × 10^−10^, the clock with a 10 MHz frequency standard at τ = 0 s would differ by about 1 mHz after 1 s. Generally, clock ADEV degrades over long-term averaging time because of frequency drift and aging [16].

Several methods have been introduced for computing ADEV to improve the results statistically. For instance, the overlapping ADEV—adopted in this study—is the most widely used method as this can provide better statistical confidence by making use of more of the data within a dataset. In some studies, the Modified ADEV (MDEV) is used to distinguish the white and flicker frequency noises [50]. ADEV values are normally plotted in log-log graphs. Further information on the different methods and their calculation formulas can be found in [51] and references therein.

**Figure 2:**
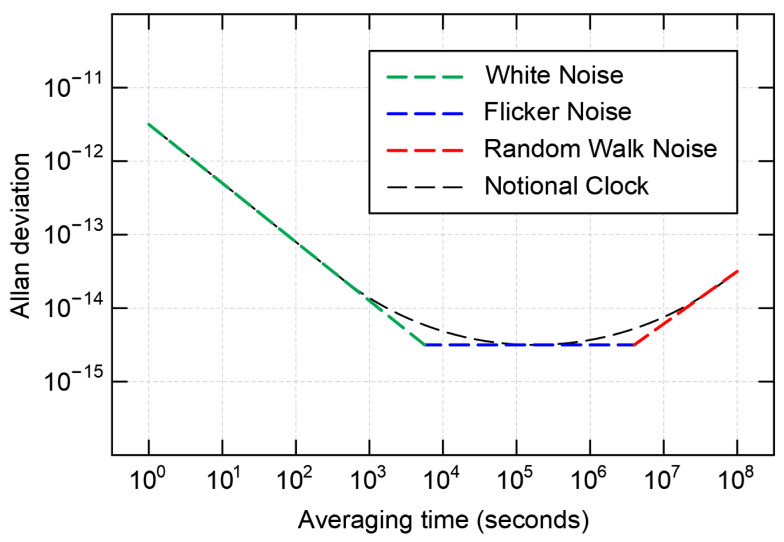
Typical dependency of clock stability against averaging time for a notional clock. The green, blue, and red lines indicate white, flicker, and random walk frequency noises, respectively. This diagram is derived from [52].

The frequency stability of most clocks can be modelled through the combination of various power-law noises [52] such as White Phase Modulation (WPM), Flicker Phase Modulation (FPM), White Frequency Modulation (WFM), Flicker Frequency Modulation (FFM), and Random Walk Frequency Modulation (RWFM). AVAR cannot separate phase modulation noises; however, it can distinguish frequency noises such as white, flicker, and random walk. Figure 2 shows typical noise behaviours on ADEV vs. Averaging time plot where the black dash curve is ADEV of a notional clock, and the green, blue, and red lines are white, flicker, and random walk frequency noise contributions, respectively.

Additionally, Table 1 provides the relationship between AVAR and power-law noises formulated in [51]. Conveniently, AVAR identifies the power-law noises differently and results in the estimation of their strengths.

## 4. Clock Stability Analysis

A clock stability analysis is carried out to compare the three successfully developed optical clocks; CROC, Iodine MTS, and Sr Lattice clock, against current GNSS satellite atomic clocks. It is important to note that the ADEV values of the optical clocks are sourced from the relevant literature [11,12,13], which presented the clock direct measurement outcomes. In contrast, we are limited to obtaining such information for the GNSS atomic clocks. Therefore, we can only deduce the clock errors indirectly from 24 h of IGS clock products with 30 s intervals to estimate the ADEV values. So, IGS clock products would necessarily incorporate other noise sources and estimation biases. On the other hand, IGS clock products are derived from a weighted ensemble of satellite clocks and the global infrastructure of monitoring stations in reference to the IGS time scale, which is determined with a two-state polynomial model driven by a mainly white noise process [53]. So, for simplicity, it is assumed that no modelling errors or reference time scale biases are found in the clock products. In total, 12 IGS clock products are used in this study spanning from 1 January 2022 to 1 December 2022 (the first day of each month in 2022). The overlapping ADEV method is used as it has the advantage of maximizing the use of all possible combinations in the data set compared to the other non-overlapping methods [51]. The overlapping ADEV, $\sigma_{y}\left(\tau \right),$ as formulated by [54], with the averaging time (τ) reads,

(1) $$\sigma_{y}\left(\tau \right)=\sqrt{\frac{1}{2\left(N-2m\right)\tau^{2}}\sum_{i=1}^{N-2m}{\left(x_{i+2m}-2x_{i+m}+x_{i}\right)}^{2}}$$

where *N* is the number of original time residual measurements, *m* is averaging factor defined as $m=\tau /\tau_{0}$, $\tau_{0}$ is the original sampling intervals (seconds), and $x_{i}$ is the *i*-th of *N* values. The averaging times (τ)  are expressed in seconds at 30, 60, 120, 240, 480, 900, 1800, 3600, 7200, 14,400, and 28,800. The 30 s and 900 s are the main interest of this study, given 30 s is the shortest averaging time estimated from the clock products, and 900 s, under which white noises drive most satellite clocks. Also, 900 s is the sampling interval of IGS Ultra-Rapid precise orbit and clock products for GNSS real-time users [55].

In light of the recent formation of four fully operational GNSS constellations, we investigated quantifying and differentiating the satellite clocks of each constellation to improve the data preparation and processing. As a result, according to the IGS clock products, there were 122 satellites in operation from the four main GNSS constellations in 2022.

We classified the satellites into different groups as per the satellite vehicle types (or generations) and estimated the total number of atomic clocks, assuming that the satellite clock types are the same in each satellite generation. As per the details in Table 2, there were 439 atomic clocks onboard GNSS satellites maintaining the continuous operation of the four constellations in 2022, 260 of which are Rubidium Atomic Clocks (Rb), 110 are Passive Hydrogen Maser Clocks (PHM), and the remaining 69 clocks are Caesium Atomic Clocks (Cs). Rb clocks are predominantly employed for GNSS satellite missions. However, it is found that different atomic clocks are assembled and employed on the same satellite together. Thus, satellite types or Pseudo-Random Noise (PRN) are referred to as the ensemble of atomic clocks in Table 2.

In terms of computational activities, ADEV values of atomic clocks assembled on 122 satellites are estimated separately for each clock product first, which are then further calculated to determine the average of all 12 clock products over 2022. Then, the averaged ADEV values of each satellite are categorised into different groups or types (see SV types in Table 2). The mean values are calculated afterwards to compare the generations of GNSS satellite clocks with the optical clocks.

## 5. Results

**Figure 3:**
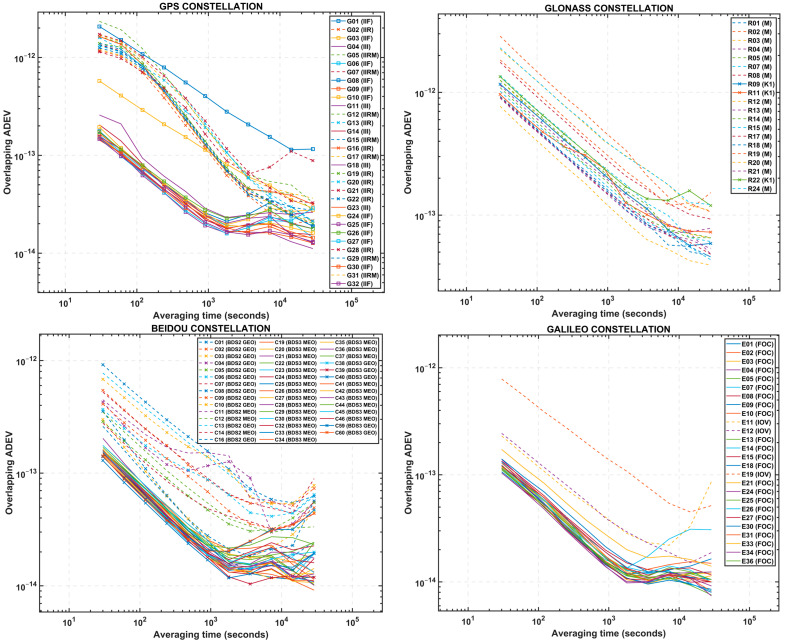
Calculated overlapping ADEV values of the satellite clocks based on IGS daily clock products with 30 s intervals in 2022. Each line denotes the ADEV values of the satellite clocks.

This section presents the clock stability analysis results. The averaged overlapping ADEV values of 12 clock products for each satellite clock, $\sigma_{y}\left(\tau \right)$ vs. averaging time, (τ) are shown on the log-log graph in Figure 3, where four separate graphs are exhibited representing the four constellations.

Looking into the GPS constellation, it is found that the new generation of Rb clocks on GPS IIF and GPS III satellites, excluding G08 and G10, have shown more than 10 times better stability yielding almost 10^−13^ at 30 s, compared to that of old Rb atomic clocks onboard GPS IIR or GPS IIR-M block satellites, which have resulted in less than 10^−12^. Furthermore, for a longer averaging time up to 900 s, the GPS IIF and GPS III satellite clocks have consistent slopes in the ADEV values driven by white noises, whereas GPS IIR and GPS IIR-M satellite clocks exhibit significant slope changes after 100 s.

Considering the Galileo and BeiDou constellations next, the ensemble of two PHM + two Rb clocks on both Galileo Full Operational Capability (FOC) and BDS3 satellites have somewhat similar slopes in the first 900 s or so, except for E07, which shows a flicker noise behaviour after 1800 s. In terms of ADEV, these clocks have shown the best stability amongst all satellite clocks at the averaging time of 30 s and 900 s reaching 10^−13^ and 10^−14^, respectively, which indicates that this unique setup of clocks has an order of magnitude better stability than their older generations of Galileo In-Orbit Verification (IOV) and BDS2 satellite clocks. Lastly, with the GLONASS constellation, no significant difference is found in the performance of M and K1 satellite clocks, given that all clock ADEV values vary around 10^−12^ and 10^−13^ at 30 s and 900 s, respectively.

**Figure 4:**
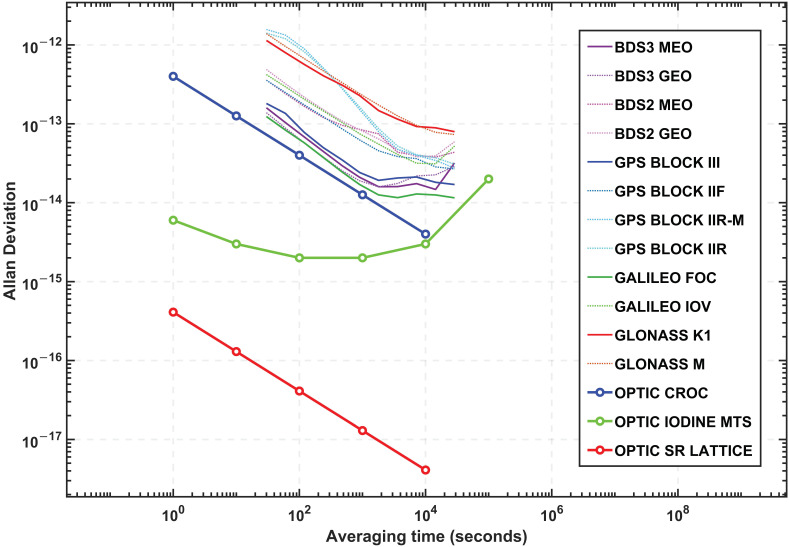
Calculated ADEV of the GNSS satellite clocks (averaged as per the satellite types) in 2022 against the CROC, Iodine MTS, and Sr Lattice Clock. The optical clock ADEV values are sourced from the existing literature [11,12,13].

The second assessment conducted is to compare the satellite clocks with the optical clocks with regard to their ADEV. For clarity on the ADEV plot, we classified and averaged the initial ADEV values previously shown in Figure 3 as per the satellite types. As detailed in Table 2, there are 12 different satellite types for the four constellations. Figure 4 presents the averaged ADEV values of the clocks (by satellite types) and the optical clocks. Among them, the ensemble of two PHM and two Rb clocks onboard Galileo FOC satellites are the most stable satellite clocks averaging 1.2 × 10^−13^ at 30 s and 1.7 × 10^−14^ at 900 s, followed by the similar setup of BeiDou BDS3 Medium Earth Orbit (MEO) clocks that have ADEV of 1.6 × 10^−13^ and 2.1 × 10^−14^ at 30 s and 900 s, respectively. The three Rb clocks on GPS Block III satellites are the most stable amongst the other GPS clocks having ADEV of 1.8 × 10^−13^ at 30 s and 2.4 × 10^−14^ after 900 s. In contrast, the three Cs clocks on GLONASS M type satellites have shown ADEV of 1.38 × 10^−12^ at 30 s and 2.4 × 10^−13^ at 900 s, which seem the least stable clocks of all four constellations overall, although the short-term stability of the clocks at 30 s is slightly better than the GPS Block IIR and IIR-M clocks with 1.41 × 10^−12^ and 1.57 × 10^−12^, respectively.

In Figure 4, the ensemble of two PHM and two Rb clocks onboard Galileo FOC satellites are the most stable satellite clocks averaging 1.2 × 10^−13^ at 30 s and 1.7 × 10^−14^ at 900 s, followed by the similar setup of BeiDou BDS3 Medium Earth Orbit (MEO) clocks that have ADEV of 1.6 × 10^−13^ and 2.1 × 10^−14^ at 30 s and 900 s, respectively. The three Rb clocks on GPS Block III satellites are the most stable amongst the other GPS clocks having ADEV of 1.8 × 10^−13^ at 30 s and 2.4 × 10^−14^ after 900 s. In contrast, the three Cs clocks on GLONASS M type satellites have shown ADEV of 1.38 × 10^−12^ at 30 s and 2.4 × 10^−13^ at 900 s, which seem the least stable clocks of all four constellations overall, although the short-term stability of the clocks at 30 s is slightly better than the GPS Block IIR and IIR-M clocks with 1.41 × 10^−12^ and 1.57 × 10^−12^, respectively. The estimated ADEV values of the satellite clocks as per the satellite types are summarised in Table 3.

**Table 3:** The estimated ADEV values of satellite atomic clocks.

Constellation	SV Type	ADEV Averaging Times										
		30 s	60 s	120 s	240 s	480 s	900 s	1800 s	3600 s	7200 s	14,400 s	28,800 s
BEIDOU	BDS2 GEO & IGSO	4.89 × 10^−13^	3.22 × 10^−13^	2.17 × 10^−13^	1.51 × 10^−13^	1.08 × 10^−13^	8.44 × 10^−14^	6.43 × 10^−14^	4.71 × 10^−14^	3.81 × 10^−14^	3.92 × 10^−14^	5.93 × 10^−14^
BEIDOU	BDS2 MEO	3.58 × 10^−13^	2.42 × 10^−13^	1.66 × 10^−13^	1.20 × 10^−13^	9.53 × 10^−14^	8.50 × 10^−14^	7.46 × 10^−14^	4.36 × 10^−14^	3.96 × 10^−14^	3.79 × 10^−14^	4.36 × 10^−14^
BEIDOU	BDS3 GEO & IGSO	1.39 × 10^−13^	8.83 × 10^−14^	5.74 × 10^−14^	3.79 × 10^−14^	2.53 × 10^−14^	1.90 × 10^−14^	1.59 × 10^−14^	1.75 × 10^−14^	2.18 × 10^−14^	2.26 × 10^−14^	2.89 × 10^−14^
BEIDOU	BDS3 MEO	1.60 × 10^−13^	1.04 × 10^−13^	6.80 × 10^−14^	4.45 × 10^−14^	2.93 × 10^−14^	2.10 × 10^−14^	1.59 × 10^−14^	1.60 × 10^−14^	1.75 × 10^−14^	1.47 × 10^−14^	3.19 × 10^−14^
GPS	Block IIF	3.57 × 10^−13^	2.49 × 10^−13^	1.74 × 10^−13^	1.23 × 10^−13^	8.58 × 10^−14^	6.25 × 10^−14^	4.55 × 10^−14^	3.85 × 10^−14^	3.62 × 10^−14^	2.85 × 10^−14^	2.68 × 10^−14^
GPS	Block III	1.81 × 10^−13^	1.36 × 10^−13^	7.76 × 10^−14^	4.98 × 10^−14^	3.47 × 10^−14^	2.42 × 10^−14^	1.92 × 10^−14^	2.06 × 10^−14^	2.11 × 10^−14^	1.81 × 10^−14^	1.70 × 10^−14^
GPS	Block IIR	1.41 × 10^−12^	1.20 × 10^−12^	8.18 × 10^−13^	4.94 × 10^−13^	2.84 × 10^−13^	1.63 × 10^−13^	8.68 × 10^−14^	5.23 × 10^−14^	4.05 × 10^−14^	3.71 × 10^−14^	3.08 × 10^−14^
GPS	Block IIR-M	1.57 × 10^−12^	1.33 × 10^−12^	8.91 × 10^−13^	5.10 × 10^−13^	2.77 × 10^−13^	1.52 × 10^−13^	7.96 × 10^−14^	4.76 × 10^−14^	3.98 × 10^−14^	3.43 × 10^−14^	2.75 × 10^−14^
GALILEO	FOC	1.24 × 10^−13^	8.33 × 10^−14^	5.73 × 10^−14^	3.73 × 10^−14^	2.44 × 10^−14^	1.70 × 10^−14^	1.26 × 10^−14^	1.16 × 10^−14^	1.29 × 10^−14^	1.25 × 10^−14^	1.15 × 10^−14^
GALILEO	IOV	4.20 × 10^−13^	2.95 × 10^−13^	2.05 × 10^−13^	1.46 × 10^−13^	1.03 × 10^−13^	7.49 × 10^−14^	5.53 × 10^−14^	4.09 × 10^−14^	3.17 × 10^−14^	3.11 × 10^−14^	5.23 × 10^−14^
GLONASS	K1	1.14 × 10^−12^	7.98 × 10^−13^	5.65 × 10^−13^	4.07 × 10^−13^	3.08 × 10^−13^	2.28 × 10^−13^	1.47 × 10^−13^	1.15 × 10^−13^	9.25 × 10^−14^	8.90 × 10^−14^	7.95 × 10^−14^
GLONASS	M	1.39 × 10^−12^	9.59 × 10^−13^	6.70 × 10^−13^	4.69 × 10^−13^	3.31 × 10^−13^	2.41 × 10^−13^	1.73 × 10^−13^	1.26 × 10^−13^	9.56 × 10^−14^	7.80 × 10^−14^	7.32 × 10^−14^

Regarding optical clocks, the Sr Lattice optical clock is the most stable optical clock with exceptional short-term stability of 4.1 × 10^−16^ at 1 s, and 4.1 × 10^−18^ over 10,000 s [13]. As opposed to the Galileo FOC satellite clocks at 30 s averaging time, the Sr Lattice optical clock is significantly better, and the corresponding ADEV value is 7.5 × 10^−17^. The second most stable optical clock is the Iodine MTS optical clock, which has also shown better short-term stability of 6 × 10^−15^ at 1 s and 2 × 10^−15^ at 900 s. Though, the steady trend in ADEV values around 3.2 × 10^−15^ over the averaging time until 10,000 s results in lower stability because the clock has seemingly experienced a flicker noise. Lastly, with the CROC, its overall stability is almost two times better than the Galileo FOC satellite clocks in the first 900 s, and it is changed thereafter to three times better stability up to 10,000 s averaging time. So, the CROC ADEV values at 30 s and 900 s are 7.3 × 10^−14^ and 1.3 × 10^−14^, respectively.

## 6. Discussion

In this section, the potentiality of optical clocks in GNSS positioning is discussed based on the research findings, the stability analysis, and the estimated clock impacts on satellite observations used for positioning. According to the stability analysis, the optical clock stability is superior to the satellite atomic clocks estimated, and therefore optical clocks could potentially surpass the current GNSS timing capability, thus positioning and navigation. For example, the most stable satellite clocks—the Galileo FOC satellite clocks—showed 1.2 × 10^−13^ at 30 s averaging time, whereas the three optical clocks; Sr Lattice, Iodine MTS, and CROC, had significantly better stability yielding 7.5 × 10^−17^, 2.8 × 10^−15^, and 7.3 × 10^−14^ respectively. Such short-term stability between 30 s and 900 s is of interest for GNSS Precise Point Positioning (PPP) real-time application users because the performance driving factor of the PPP application is IGS precise orbit and clock products. For longer averaging times over 900 s, the stability of three optical clocks is still better, outweighing the satellite clocks by orders of magnitudes.

Regarding use cases, clock long-term stability is pertinent in predicting satellite clock errors for broadcast navigation messages, which GNSS Single Point Positioning (SPP) application, for example, is reliant on. GPS and GLONASS navigation messages are uploaded daily from the ground segments to the satellites, where a set of ephemerides data is transmitted to receivers every 120 min and 30 min, respectively. In comparison, Galileo and BeiDou constellations upload their navigation messages in a much shorter period of 10 and 60 min, respectively. So, the averaging time for a clock prediction must be limited in line with the navigation message uploading intervals [7] because the predicted clock information is refreshed upon the uploads.

**Figure 5:**
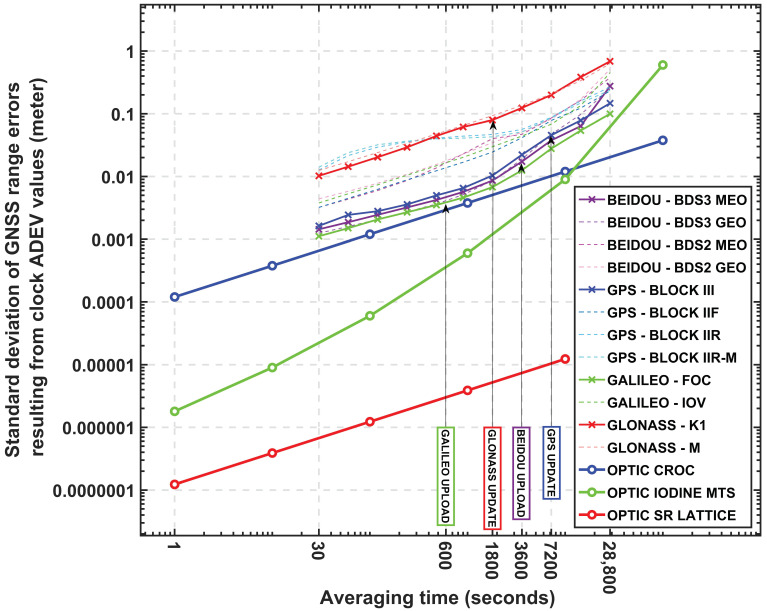
The standard deviation of GNSS range errors resulting from the clock ADEV values presented with different colours of lines. The upload intervals of navigation data for each constellation are highlighted additionally.

Following that, simple calculations have been completed here to gauge the clock ADEV contribution (solely) to GNSS range measurement errors. As formulated by [7], assuming the clock drift and drift rate are perfectly determined, the standard deviation of range errors—SD(τ)—over different averaging times resulting from clock ADEV values can be estimated as

(2) $$SD\left(\tau \right)=c\times \sigma_{y}\left(\tau \right)\times \tau$$

where c is the speed of light. Figure 5 displays the calculated standard deviations of range errors as a function of averaging time for the GNSS satellite and optical clocks. The typical upload intervals (first update) of broadcast navigation messages are also highlighted in the figure.

At first glance, the overall standard deviation of clock prediction from the GNSS satellite clocks is less than 1 m over eight hours of averaging time, whereas the optical clock contributions are under a decimetre to millimetre level. Further in detail, at 30 s averaging time, where PPP real-time application is concerned, the optical clocks may reduce the prediction errors to 1 mm level from 14 mm, which is the greatest of all. SPP application users, in contrast, can expect less than a centimetre level error from the optical clocks, which is approximately a 10 times better result than the Galileo FOC satellite clocks.

Furthermore, an individual constellation’s clock prediction errors in the corresponding navigation message intervals are discussed based on the estimated stability of the satellite clocks. For example, a similar setup of Galileo FOC and BeiDou BDS3 satellite clocks will contribute an error of a few millimetres to centimetres to the clock prediction within their navigation data upload intervals. Whereas the stability of GLONASS and GPS satellite clocks will have clock prediction errors of less than a decimetre over 1800 s and 7200 s, respectively. The three optical clocks, for comparison, will result in millimetre to micrometre levels of errors in the window of upload intervals of all constellations ranging from 600 s to 7200 s, despite the fact that the stability of the Iodine MTS and CROC clock have degradation after 10,000 s.

However, considering the clock technology readiness level (TRL), which describes the maturity of technologies and Size, Weight, and Power (SWAP) parameters, these three clocks may require further enhancement. The Sr Lattice optical clock, for instance, needs to be miniaturised to meet the satellite payload requirement, which is one of the critical features in choosing an appropriate clock, besides stability, for a reliable, long-term satellite operation [15,56]. The current size and weight of the Sr Lattice optical clock are approximately 968 L and 250 kg, respectively [13]. It was also mentioned that these parameters could feasibly be reduced to 500 L and 100 kg. The Galileo PHM clock’s parameters, in contrast, are 40 L and 25 kg [15]. The Iodine MTS and CROC optical clocks, on the other hand, have a much more compact setup occupying 40 L and 15 L, respectively, which allows the clocks to meet the SWAP requirements. Even the Iodine MTS clock was flown successfully on a sounding rocket to test its maturity and robustness [41,42]. But the stability of both clocks degrades after 10,000 s due to the cavity reference on the Iodine MTS, and the out-of-loop measurements of the cell temperature and laser power of the CROC.

## 7. Conclusions

This paper overviews the current development of optical clocks in the PNT. Due to the emerging optical clock technology and the ongoing necessity for maintaining or improving GNSS capability, the potential contribution of optical clocks to GNSS positioning is discussed based on the literature review and the stability analysis conducted.

Throughout the development of timing devices, optical clocks have been the most stable to date, achieving the ultimate uncertainty of 10^−18^ and beyond [4,28], which exceeds the preceding atomic clocks by two orders of magnitude in performance. The critical improvement of optical clocks was the source of the oscillator—that is, an ultra-stable laser emitting optical transitions. In contrast, a typical atomic clock configuration has a quartz crystal oscillator generating microwaves.

In terms of clock characterisation, we briefly described clock stability, noise components, ADEV measures, and SWAP parameters. It was noted that these characteristics are crucial in evaluating any atomic or optical clocks. Furthermore, in some cases, like satellite missions, both ADEV and SWAP requirements are essential because spacecraft payload criteria are thoroughly considered, whereas for clock-based applications on the Earth, especially in metrology, the SWAP requirement is less essential.

With the clock stability analysis, three successfully developed optical clocks, Iodine MTS, CROC, and Sr lattice, were compared to the current GNSS satellite atomic clocks, 439 atomic clocks, onboard 122 satellites of the four main constellations to discuss the optical clock potentiality. Amongst the satellite clocks, the ensemble of two PHM and two Rb atomic clocks onboard Galileo FOC satellites were the most stable atomic clocks with ADEV of 1.2 × 10^−13^ at 30 s and 1.7 × 10^−14^ at 900 s. Whereas for the optical clocks, the Sr Lattice optical clock was the most stable having 7.5 × 10^−17^ and 1.4 × 10^−17^ stability over 30 s and 900 s, respectively—which showed a few orders of magnitude better stability than the Galileo FOC satellite clocks.

Lastly, the optical clock potentiality in GNSS positioning is discussed by evaluating the clock stability contributions to range errors for GNSS PPP real-time and SPP applications. With the PPP application, optical clocks could decrease the clock contribution to the range errors to 1 mm over 30 s, assuming the clock drift and drift rates are flawlessly determined. Regarding the SPP application, which relies on broadcast navigation messages, users can expect 10 times better errors than the Galileo FOC satellite clocks. But considering the clock TRL and SWAP parameters, the optical clocks have not matured yet. However, in the authors’ view, the current development of optical clocks is auspicious in GNSS positioning given that its primary purpose of timekeeping is superb; nevertheless, the technology needs to mature further before being deployed on GNSS satellites.

## Figures and Tables

**Table 1:** The relationship between AVAR and clock power-law noises [51].

Power-Law Noise	Coefficient	Allan Variance Relationship
White Phase Noise (WPN)	$h_{2}$	$\frac{4\pi^{2}\tau^{2}\sigma_{y}^{2}\left(\tau \right)}{3f_{h}}$
Flicker Phase Noise (FPN)	$h_{1}$	$\frac{4\pi^{2}\tau^{2}\sigma_{y}^{2}\left(\tau \right)}{1.038+3ln(2\pi f_{h}\tau )}$
White Frequency Noise (WFN)	$h_{0}$	$2\pi^{2}\sigma_{y}^{2}\left(\tau \right)$
Flicker Frequency Noise (FFN)	$h_{-1}$	$\frac{\sigma_{y}^{2}\left(\tau \right)}{2ln\left(2\right)}$
Random Walk Frequency Noise (RWFN)	$h_{-2}$	$\frac{3\sigma_{y}^{2}\left(\tau \right)}{2\pi^{2}\tau }$

**Table 2:** Total atomic clocks onboard GNSS satellites in 2022.

Const.	SV Type	PRN	Clocks
GPS	Block IIR	G02 G13 G16 G19 G20 G21 G22 G28	3 Rb
GPS	Block IIR-M	G05 G07 G12 G15 G17 G29 G31	3 Rb
GPS	Block IIF	G01 G03 G06 G08 G09 G10 G24 G25 G26 G27 G30 G32	2 Rb + 1 Cs
GPS	Block III	G04 G11 G14 G18 G23	3 Rb
GLONASS	M	R01 R02 R03 R04 R05 R07 R08 R12 R13 R14 R15 R17 R18 R19 R20 R21 R24	3 Cs
GLONASS	K1	R09 R11 R22	2 Cs + 2 Rb
GALILEO	IOV	E11 E12 E19	2 PHM + 2 Rb
GALILEO	FOC	E01 E02 E03 E04 E05 E07 E08 E09 E10 E13 E14 E15 E18 E21 E24 E25 E26 E27 E30 E31 E33 E34 E36	2 PHM + 2 Rb
BEIDOU	BDS2 MEO	C11 C12 C14	4 Rb
BEIDOU	BDS2 GEO & IGSO	C01 C02 C03 C04 C05 C06 C07 C08 C09 C10 C13 C16	4 Rb
BEIDOU	BDS3 MEO	C19 C20 C21 C22 C23 C24 C25 C26 C27 C28 C29 C30 C32 C33 C34 C35 C36 C37 C41 C42 C43 C44 C45 C46	2 PHM + 2 Rb
BEIDOU	BDS3 GEO&IGSO	C38 C39 C40 C59 C60	2 PHM + 2 Rb

## Data Availability

The IGS clock products were provided by GeoForschungsZentrum Postdam (GFZ) at https://cddis.nasa.gov/archive/gnss/products/mgex/ (accessed on 3 March 2023). Whereas the optical clock ADEV values were sourced from the literature referenced in this study.

## References

[1] Panfilo G., Arias F. (2019). The Coordinated Universal Time (UTC). Metrologia.

[2] Tavella P., Petit G. (2020). Precise time scales and navigation systems: Mutual benefits of timekeeping and positioning. Satell. Navig..

[3] Ludlow A.D., Boyd M.M., Ye J., Peik E., Schmidt P.O. (2015). Optical atomic clocks. Rev. Mod. Phys..

[4] Brewer S.M. (2021). Frequency ratio measurements at 18-digit accuracy using an optical clock network. Nature.

[5] U.S. Department of Transportation What Is Positioning, Navigation and Timing (PNT)?. https://www.transportation.gov/pnt/what-positioning-navigation-and-timing-pnt.

[6] Morton Y.J., van Diggelen F., Spilker J.J., Bradford W.P., Sherman L., Grace G. (2021). Position, Navigation, and Timing Technologies in the 21st Century: Integrated Satellite Navigation, Sensor Systems, and Civil Applications—Set.

[7] Montenbruck O., Steigenberger P., Hauschild A. Comparing the ‘Big 4′—A User’s View on GNSS Performance. Proceedings of the 2020 IEEE/ION Position, Location and Navigation Symposium (PLANS).

[8] Hauschild A., Montenbruck O., Steigenberger P. (2013). Short-term analysis of GNSS clocks. GPS Solut..

[9] Teunissen P., Montenbruck O. (2017). Springer Handbook of Global Navigation Satellite Systems.

[10] Phelps G., Lemke N., Erickson C., Burke J., Martin K. (2018). Compact Optical Clock with 5 × 10^−13^ Instability at 1 s. Navigation.

[11] Martin K.W., Phelps G., Lemke N.D., Bigelow M.S., Stuhl B., Wojcik M., Holt M., Coddington I., Bishop M.W., Burke J.H. (2018). Compact Optical Atomic Clock Based on a Two-Photon Transition in Rubidium. Phys. Rev. Appl..

[12] Schuldt T., Gohlke M., Oswald M., Wüst J., Blomberg T., Döringshoff K., Bawamia A., Wicht A., Lezius M., Voss K. (2021). Optical clock technologies for global navigation satellite systems. GPS Solut..

[13] Origlia S., Pramod M.S., Schiller S., Singh Y., Bongs K., Schwarz R., Al-Masoudi A., Dörscher S., Herbers S., Häfner S. (2018). Towards an optical clock for space: Compact, high-performance optical lattice clock based on bosonic atoms. Phys. Rev. A.

[14] Batori E., Almat N., Affolderbach C., Mileti G. (2021). GNSS-grade space atomic frequency standards: Current status and ongoing developments. Adv. Space Res..

[15] Jaduszliwer B., Camparo J. (2021). Past, present and future of atomic clocks for GNSS. GPS Solut..

[16] Schmittberger B.L., Scherer D.R. (2021). A Review of Contemporary Atomic Frequency Standards. arXiv.

[17] Michalak G., Glaser S., Neumayer K.H., König R. (2021). Precise orbit and Earth parameter determination supported by LEO satellites, inter-satellite links and synchronized clocks of a future GNSS. Adv. Space Res..

[18] Giorgi G., Kroese B., Michalak G. Future GNSS constellations with optical inter-satellite links. Preliminary space segment analyses. Proceedings of the 2019 IEEE Aerospace Conference.

[19] Petit G., Jiang Z. (2007). GPS All in View time transfer for TAI computation. Metrologia.

[20] Poli N., Oates C.W., Gill P., Tino G.M. (2013). Optical atomic clocks. Riv. Nuovo Cim..

[21] Sharma L., Rathore H., Utreja S., Neelam, Roy A., De S., Panja S. (2020). Optical Atomic Clocks for Redefining SI Units of Time and Frequency. MĀPAN J. Metrol. Soc. India.

[22] Lodewyck J. (2019). On a definition of the SI second with a set of optical clock transitions. Metrologia.

[23] McGrew W.F., Zhang X., Leopardi H., Fasano R.J., Nicolodi D., Beloy K., Yao J., Sherman J.A., Schäffer S.A., Savory J. (2019). Towards the optical second: Verifying optical clocks at the SI limit. Optica.

[24] Terrien J. (1968). News from the International Bureau of Weights and Measures. Metrologia.

[25] Taylor B.N., U.S. Department of Commerce (2008). The International System Of Units (SI).

[26] Riehle F., Gill P., Arias F., Robertsson L. (2018). The CIPM list of recommended frequency standard values: Guidelines and procedures. Metrologia.

[27] Hinkley N., Sherman A., Phillips N.B., Schioppo M., Lemke N.D., Beloy K., Pizzocaro M., Oates C.W., Ludlow A.D. (2013). An atomic clock with 10^−18^ instability. Science.

[28] McGrew W.F., Zhang X., Fasano R.J., Schäffer S.A., Beloy K., Nicolodi D., Brown R.C., Hinkley N., Milani G., Schioppo M. (2018). Atomic clock performance enabling geodesy below the centimetre level. Nature.

[29] Bloom B.J., Nicholson T.L., Williams J.R., Campbell S.L., Bishof M., Zhang X., Zhang W., Bromley S.L., Ye J. (2014). An optical lattice clock with accuracy and stability at the 10–18 level. Nature.

[30] Zhang S., Zhang X., Cui J., Jiang Z., Shang H., Zhu C., Chang P., Zhang L., Tu J., Chen J. (2017). Compact Rb optical frequency standard with 10^−15^ stability. Rev. Sci. Instrum..

[31] Lemke N.D., Martin K.W., Beard R., Stuhl B.K., Metcalf A.J., Elgin J.D. (2022). Measurement of Optical Rubidium Clock Frequency Spanning 65 Days. Sensors.

[32] Hänsch T.W. (2006). Nobel Lecture: Passion for precision. Rev. Mod. Phys..

[33] Holzwarth R., Udem T., Hänsch T.W., Knight J.C., Wadsworth W.J., Russell P.S.J. (2000). Optical Frequency Synthesizer for Precision Spectroscopy. Phys. Rev. Lett..

[34] Fortier T., Baumann E. (2019). 20 years of developments in optical frequency comb technology and applications. Commun. Phys..

[35] Cao J., Zhang P., Shang J., Cui K., Yuan J., Chao S., Wang S., Shu H., Huang X. (2017). A compact, transportable single-ion optical clock with 7.8 × 10^−17^ systematic uncertainty. Appl. Phys. B.

[36] Koller S.B., Grotti J., Vogt S., Al-Masoudi A., Dörscher S., Häfner S., Sterr U., Lisdat C. (2017). Transportable Optical Lattice Clock with 7 × 10^−17^ Uncertainty. Phys. Rev. Lett..

[37] Newman Z.L., Maurice V., Drake T., Stone J.R., Briles T.C., Spencer D.T., Fredrick C., Li Q., Westly D., Ilic B.R. (2019). Architecture for the photonic integration of an optical atomic clock. Optica.

[38] Zhou W., Cahill J., Ni J.H., Deloach A., Cho S.-Y., Anderson S., Mahmood T., Sykes P., Sarney W.L., Leff A.C. (2021). Developing a chip-scale optical clock. Opt. Eng..

[39] Ghent University An Optical Atomic Clock on a Chip Unleashes a Revolution in Time Registration. https://www.ugent.be/voor-organisaties-en-bedrijven/en/news-events/news/optical-atomic-clock-on-a-chip-unleashes-a-revolution-in-time-registration.

[40] Schiller S., Gorlitz A., Nevsky A., Alighanbari S., Vasilyev S., Abou-Jaoudeh C., Mura G., Franzen T., Sterr U., Falke S. The space optical clocks project: Development of high-performance transportable and breadboard optical clocks and advanced subsystems. Proceedings of the 2012 European Frequency and Time Forum.

[41] Schkolnik V., Döringshoff K., Gutsch F.B., Oswald M., Schuldt T., Braxmaier C., Lezius M., Holzwarth R., Kürbis C., Bawamia A. (2017). JOKARUS—Design of a compact optical iodine frequency reference for a sounding rocket mission. EPJ Quantum Technol..

[42] Döringshoff K., Gutsch F.B., Schkolnik V., Kürbis C., Oswald M., Pröbster B., Kovalchuk E.V., Bawamia A., Smol R., Schuldt T. (2019). Iodine Frequency Reference on a Sounding Rocket. Phys. Rev. Appl..

[43] Safronova M.S., Budker D., Demille D., Kimball D.F.J., Derevianko A., Clark C.W. (2018). Search for new physics with atoms and molecules. Rev. Mod. Phys..

[44] Denker H., Timmen L., Voigt C., Weyers S., Peik E., Margolis H.S., Delva P., Wolf P., Petit G. (2018). Geodetic methods to determine the relativistic redshift at the level of 10^−18^ in the context of international timescales: A review and practical results. J. Geod..

[45] Mehlstäubler T.E., Grosche G., Lisdat C., Schmidt P.O., Denker H. (2018). Atomic clocks for geodesy. Rep. Prog. Phys..

[46] Müller J., Wu H. (2020). Using quantum optical sensors for determining the Earth’s gravity field from space. J. Geod..

[47] Takamoto M., Ushijima I., Ohmae N., Yahagi T., Kokado K., Shinkai H., Katori H. (2020). Test of general relativity by a pair of transportable optical lattice clocks. Nat. Photonics.

[48] Pizzocaro M., Sekido M., Takefuji K., Ujihara H., Hachisu H., Nemitz N., Tsutsumi M., Kondo T., Kawai E., Ichikawa R. (2021). Intercontinental comparison of optical atomic clocks through very long baseline interferometry. Nat. Phys..

[49] Nicholson T.L., Campbell S.L., Hutson R.B., Marti G.E., Bloom B.J., McNally R.L., Zhang W., Barrett M.D., Safronova M.S., Strouse G.F. (2015). Systematic evaluation of an atomic clock at 2 × 10^−18^ total uncertainty. Nat. Commun..

[50] Allan D.W., Barnes J.A. A Modified “Allan Variance” with Increased Oscillator Characterization Ability. Proceedings of the Thirty Fifth Annual Frequency Control Symposium.

[51] William R., David H. (2008). Handbook of Frequency Stability Analysis.

[52] Howe D.A., Allan D.U., Barnes J.A. Properties of Signal Sources and Measurement Methods. Proceedings of the Thirty Fifth Annual Frequency Control Symposium.

[53] Senior K.L., Ray J.R., Beard R.L. (2008). Characterization of periodic variations in the GPS satellite clocks. GPS Solut..

[54] Allan D., Hellwig H., Kartaschoff P., Vanier J., Vig J., Winkler G.M.R., Yannoni N.F. Standard terminology for fundamental frequency and time metrology. Proceedings of the 42nd Annual Frequency Control Symposium.

[55] IGS (2020). IGS Products. https://igs.org/products/#about.

[56] Hofmann-Wellenhof B., Lichtenegger H., Wasle E. (2008). GNSS—Global Navigation Satellite Systems GPS, GLONASS, Galileo, and More.

